# Lectures on Subjects Connected with Clinical Medicine: Comprising Diseases of the Heart

**Published:** 1847-05

**Authors:** 


### REVIEWS.

---

Art. IV.—*Lectures on Subjects connected with Clinical Medicine: comprising Diseases of the Heart.* By P. M. Latham, M.D., Fellow of the Royal College of Physicians; Physician Extraordinary to the Queen, and late Physician to St. Bartholomew's Hospital. 8vo. pp. 365. Philadelphia: Ed. Barrington & Geo. D. Haswell. 1847. [Bell's Medical Library.]

Dr. Latham's name is already familiar to our readers as that of a correct writer, and an able and experienced author. His former volume of lectures on clinical medicine, introduced from abroad through Bell's Select Medical Library, was everywhere received with merited favor; and we feel certain, that the present volume is destined to a much wider popularity.

The diseases of the heart are among the most interesting that we are called upon to treat, and are those wherein the practical usefulness, not to say the absolute necessity, of a knowledge of auscultatory signs is most clearly shown. The stethoscope has disclosed to us that what was considered rare, is frequent; what was thought accidental, is constant; and what was known for the most part as a result and irremediable, is now for the most part obvious at its beginning, and while yet within the power of art. Until it was brought to bear successfully upon these affections, they were little else than matters of curiosity. Specimens of diseased hearts were collected and preserved in cabinets, and were gazed at by the curious, just as fossils were wont to be kept and looked at and gazed at in the cabinet of the naturalist. Or they garnished museums of morbid anatomy, as trophies of the dead-house, speaking of pain and anguish and mortal suffering, though none could tell how or when; just as in the museum of the antiquary battered shields, broken lances, and cleft helmets, dug from some unrecorded battle-field, speak of the "drums and tramplings" of contending hosts, though none can tell how or when. Auscultation brought to the consideration of these diseases "a new light, and a new interest." Yet for a long time the practical benefit was small. It required the labor of years and of many minds—of such minds as gather up unconsidered trifles in the way of knowledge—to attain any profitable result.

"And at length the mark has been hit, and the prize has been won. For now there is no truth experimentally more certain than this, that auscultatory signs above all others, and oftentimes before all others, and oftentimes in the place of all others, may be safely trusted to declare the beginning and the augment, the decline and the cessation of acute inflammation in those structures of the heart, which are especially, if not solely, obnoxious to it; and that the same signs may be confidently appealed to as guides, by which to choose the remedy, and apportion its quantity and regulate its force, and continue or discontinue its application."

Dr. Latham, after a very lucid account of the sounds, impulses, and resonances of the heart, the mode in which they become signs of its disease, and the value of these signs, proceeds to the discussion of inflammation of the heart, and its connection with acute rheumatism. It is here we shall begin our quotations, as it is a subject of practical interest and one which we hope to compress within the necessary limits. He has found inflammation of the heart, in the form of endocarditis and pericarditis, separately or together, much more frequent in connection with acute rheumatism than it is generally supposed to be.

"Between the years 1836 and 1840, both inclusive, there occurred under my care at St. Bartholomew's Hospital 136 cases of acute rheumatism:

"Of these 136 patients 75 were males, and 61 were females; of the 75 males the heart was affected in 47, and unaffected in 28.

"Of the 47, the seat of disease was the endocardium alone in 30; the pericardium alone in 3; and both the endocardium and pericardium in 7. And, while the heart was undoubtedly affected in 7 others, the exact seat of its disease was uncertain.

"Of the whole number of males in whom the heart was thus variously affected, 3 died. And in these 3 the pericardium and the endocardium were both inflamed.

"Of the 61 females, the heart was affected in 43, and unaffected in 18.

"Of the 43, the seat of disease was the endocardium alone in 33; the pericardium alone in 4; and both endocardium and pericardium in 4; and the exact seat of the cardiac disease was doubtful in 2.

"Of the whole number of females in whom the heart was thus variously affected none died.

"The account of males and females taken together will stand thus:—

| | |
|---|---|
| Cases of acute rheumatism - - | 136 |
| Heart exempt in - - - | 46 |
| Heart affected in - - - | 90 |
| Seat of disease in the heart:— | |
| Endocardium alone in - - | 63 |
| Pericardium alone in - - | 7 |
| Endocardium and pericardium in | 11 |
| Doubtful in - - - - - | 9 |

Deaths 3. In all of whom both endocardium and pericardium were affected.

"Here are momentous facts, which go (I suspect) a good deal beyond the ordinary notions entertained by medical men of this matter. It is believed that among the sufferers of acute rheumatism, an individual now and then unluckily has his heart inflamed. The thing is looked upon as an accident which, if not very rare, yet is not very common. But it appears, from the event, not of a dozen or twenty cases merely, but of a number large enough to furnish the measure of what naturally belongs to the disease, that as many as two-thirds of those who have acute rheumatism also suffer inflammation of the heart.

"Further, the pericardium is popularly regarded as the special and most frequent seat of the inflammation which takes its rise from acute rheumatism. But it appears from cases sufficiently numerous, that endocarditis occurs nine times in acute rheumatism, for pericarditis once; that simple endocarditis constitutes more than two-thirds of all rheumatic cardiac affections, and simple pericarditis only one-thirteenth; and that pericarditis is more frequently found in combination with endocarditis than alone."

The lungs are also liable to suffer in acute rheumatism, and in rheumatic inflammation of the heart. It may be a bronchitis, a pneumonia, or a pleurisy, and in all cases puts on a serious character.

"Of the 136 cases of acute rheumatism which form the basis of our inquiry, inflammation of the lungs was found in 24. Here the proportion is about 1 in 5½. But *how* were these 24 cases distributed? What proportion of them occurred where the heart was unaffected, and what proportion where the heart was inflamed? And, again, what proportion, where the inflammation was of the endocardium alone; what, where it was of the pericardium alone; and what, where it was of the endocardium and pericardium simultaneously?

"Of the 46 cases of acute rheumatism in which the heart was unaffected, the lungs were inflamed in 5. Here the proportion is as one to nine. But of the 90 cases in which the heart was inflamed, the lungs were also inflamed in 19. Here the proportion is more than one in five.

"The 19 instances of inflammation of the lungs were distributed among these 90 cases of inflammation of the heart in different proportions, according to the part of the organ affected.

"Of the 63 cases of endocarditis the lungs were inflamed in 7. Here the proportion is as one to nine.

"Of the 7 cases of pericarditis the lungs were inflamed in 4. Here the proportion is more than one-half.

"Of the 11 cases of endocarditis and pericarditis simultaneously, the lungs were inflamed in 8. Here the proportion is more than two-thirds.

"What can we, or what can we not, conclude from this enumeration of facts? What general truths do they declare?

"We cannot conclude, from inflammation of the lungs being found in one case of acute rheumatism out of nine, that acute rheumatism has any strong natural tendency to inflame the lungs. Neither can we conclude, from its being found in one case of rheumatic endocarditis out of nine, that rheumatic endocarditis has a special natural connexion with inflammation of the lungs. The probability of inflammation of the lungs arising out of acute rheumatism is small; and the probability is not at all augmented by its alliance with endocarditis. For in acute rheumatism, inflammation of the lungs does not occur more frequently when the endocardium is inflamed, than when the heart is entirely exempt from disease.

"We find it to be between inflammation of the pericardium and inflammation of the lungs, and between inflammation of the endocardium and pericardium occurring simultaneously in the same subject, and inflammation of the lungs, that frequent coincidence seems to establish a natural connexion.

"That inflammation of the heart which is least perilous in itself is least liable to have its danger augmented by an union with inflammation of the lungs, viz: endocarditis; while that which is most perilous in itself is most frequently accompanied by inflammation of the lungs, adding immensely to its danger, viz: simple pericarditis, or endocarditis combined with pericarditis."

Endocarditis and pericarditis being the same in essence (inflammation), often occurring together in the same subject, and belonging to the same pathological condition, are to be treated by the same remedies. The treatment is either *preventive* or *curative*. The former consists in the proper man agement of the general pathological state out of which they arise. Acute rheumatism, it is well known, is successfully treated by the most various and opposite remedies. Some look entirely to the vascular system, and depend upon blood-letting; some to the nervous system, and rely on opium; some to the stomach and bowels and liver, and trust to calomel and purgatives.

"At the first view all this looks very strange. The cure, or seeming cure, of the same disease by different remedies, even by remedies which in their mode of operation have nothing in common, appears like luck or accident. At the first view it may shake one's faith in physic a little, and may a little excuse the pleasantry of some who choose to hint, that Nature is our best friend after all; for do what we will, she brings things to a prosperous issue in spite of our blind inference.

"But without disparaging the part that Nature plays, I here see no fair subject of ridicule, and no fair reason for distrust of methods of rational treatment. The first maxim of all rational practice is, that Nature is supreme; the next, that Nature is obsequious. The end, whether bad or good, death or recovery, and every step and stage conducive to it, are the unquestionable work of Nature. But Nature, in all her powers and operations, allows herself to be led, directed, and controlled. And to lead, direct, or control for purposes of good, this is the business of the physician. But how to do it best, he has to exercise a choice of modes and means in every case, which, though never exempt from the possibility of error, becomes less fallible by the teaching of experience.

"This choice leads, and always will lead, to diversity of practice, which no way disparages, but rather tends to enlarge and to enrich the resources of our art."

Of these three modes, the author thinks that by opium better than that by bleeding, and that by calomel and purgatives better than either. His discussion of these we must omit, although it is both interesting and able. We cannot forbear, however, giving the following quotation in reference to blood-letting:

"Now, one chief hazard of large bleeding is from the shock it is apt to communicate to the nervous system; and it is this which we should especially seek to avoid, when we employ venesection in acute rheumatism. Venesection is often needed; needed for what no other remedy can perform towards the cure, and therefore not to be omitted. It is needed especially to abate high vascular action. But no such thing must be thought of as bleeding, and bleeding, until the large pulse becomes small, and the hard pulse soft. For, to bring about this, blood must be let flow to a terrific amount. Fulness and hardness of pulse are indeed express characteristics of acute rheumatism, rising to superlative degrees, and enduring pertinaciously, and resisting stubbornly the power of remedies to pull them down. To abolish them at once or speedily in the severer cases, is hardly possible by any counteracting means which medicine can safely employ. They are above a match for them all, and they *will* endure their time. They are above a match even for venesection, unless it be pushed with a desperate hand, careless and reckless of new dangers to life."

After a full examination of these methods, both theoretically and practically, Doct. Latham insists that a mixed treatment—venesection, opium, and calomel—is the best for acute rheumatism, and the safest as a preventive of the cardiac affections.

"The practice which chooses and follows a single indication, and chooses and trusts to a single remedy, is indeed a plain and intelligible, but a harsh and subduing, practice. In cases of ordinary severity, if the entire disease is to be effectually reached and counteracted through the bloodvessels alone, a single venesection of large amount would be needed, or a venesection of smaller amount once or twice repeated. If through the nerves alone, four or five grains of opium would be needed in each 24 hours. If through the abdominal viscera alone, a large dose of calomel and a draught of senna and salts would be required night and morning for three or four successive days.

"Here is much violence done and felt as the price of success. Venesection, single-handed, to do its work successfully, must strike with the violence that shocks, opium with the violence that oppresses, calomel and purgatives with the violence that hurts and irritates. It cannot be otherwise. If one remedy is to do all, it must be heavily charged and resolutely driven home to its purpose.

"But each remedy may be charged with less force, if one be made auxiliary to the other. Bloodvessels, and nerves, and abdominal viscera may be severally spared the shock, the oppression, and the pain, if they are subjected simultaneously to their several remedies. Thus, in cases of ordinary severity, a single moderate venesection instead of several, or instead of one of large amount, two grains of opium distributed over twenty-four hours instead of four or five grains, moderate doses of calomel followed by purgatives instead of very large doses given and repeated for three or four successive nights and mornings, comprise a treatment powerful enough, and always safe, and generally successful, and not painfully felt. Perhaps it would come pretty near the truth to say, that two-thirds less of blood-letting, two-thirds less of opium, two thirds less of calomel and purgatives, are needed when they are all made confederate for the cure of acute rheumatism, than when any one of them is employed alone.

"In several cases, however, the proportion of the remedies to each other would vary. Sometimes more, sometimes less, blood-letting would be called for, and often none at all. So, too, more or less opium, and more or less calomel and aperients.

"I have remarked of the effect of blood-letting in acute rheumatism, that it belongs to it rather to render the disease more curable by other remedies than to cure it itself. Blood-letting, therefore, properly takes the lead of other remedies in point of time. For if it be necessary, the whole treatment must tarry, and other remedies come short of the good of which they are capable, until it is performed. On the first view, then, of the patient, the immediate question is: Should he be bled? and if so, to what amount? This question it would be foolish and dangerous to pretend to settle anywhere but in the wards of a hospital, and with the very patient before you, and your finger upon his pulse. Well! and what then? Why! then, if you judge that there is more force of circulation than calomel and purgatives operating upon the bowels, aided by the soothing effects of opium upon the nervous system, will be able to abate, you may bleed.

"This is the best direction I can give. But, be it the best and wisest that can be given, it must be utterly useless, nevertheless, except to those who are or shall be constantly busied about the sick. For by this direction, whether to bleed or not to bleed is made to wait upon a judgment which must first be formed upon two points. And these two points nothing but the most constant bedside experiments can make sure of. They are these—the exact force of the circulation, in a particular instance, to be ascertained by the pulse; and the probable power of calomel, and purgatives, and opium to reduce it, to be estimated by the ordinary effects of the same remedies.

"I do not wish to exaggerate the difficulties of medical practice; neither do I wish to conceal them. I am sure you will never surmount them, unless you first feel and acknowledge them. And some practical experience is needed even for this.

"When from the pulse I have considered venesection necessary to bring down the circulation, the loss of between twelve and sixteen ounces of blood has generally been enough to answer the purpose in view; and the venesection has seldom been repeated.

"The opium, and calomel, and purgatives I have been accustomed to give in combination thus:—With calomel administered at night, according to its quantity, I have united more or less of opium. To ten grains of calomel I have added one grain of opium; or to five of calomel I have added half a grain, continuing to give them together in the same proportion, night after night, as long as they were needed. Then, on each succeeding day, when a large purgation of the bowels has been duly obtained, I have still given the opium alone, or with saline draughts, in doses of half or one-third of a grain, every five or six hours. And thus, with the larger quantity at night, and the smaller quantities during the day, about two grains of opium have been commonly taken in the course of twenty-four hours.

"Here, then, the vascular system, and the nervous system and the abdominal viscera are all at the same time made to feel sensibly the impression of the remedy, but none of them is subdued by it. And while blood-letting, and opium, and calomel with purgatives are all made confederate for the cure of the disease, none of them is given in excess.

"Now, I do not pretend to say, that this is just the measure, and just the relative proportion in which these several remedies need always to be employed for the cure of acute rheumatism. There are circumstances which would require them to be varied. But, apart from the patient, they cannot be represented intelligibly. As of venesection, so of these other remedies, after the propriety of their use is already understood, the skill of using them remains to be learnt; the skill which sees when to give a little more, and when a little less, oftener or seldomer—when to bear heavily or lightly on the bloodvessels, when heavily or lightly on the nerves—and when to obtain larger or smaller purgation of the abdominal viscera. This is the skill which cures diseases and saves lives. And no man ever had it, who did not obtain it from his own self-teaching amid the emergencies of actual practice."

Colchicum cannot be trusted in the severer cases, but may be in the milder ones. Even in the latter it is slow, and does no good unless it acts "in the first degree of its poisoning power," viz: purging smartly and painfully. Still the author employs it in special emergencies, with a trust and confidence he has in no other remedy.

"When by venesection and by opium and calomel with purgatives, excess of vascular action, and fever and pain and swelling are abated, yet none of them are entirely abolished, but all still linger; or when pain and swelling do not subside at all in proportion to the abatement of vascular action and fever, which are considerably reduced, then I invoke the aid of colchicum, and give twenty or five-and-twenty minims of the wine of the seeds or the root, twice or thrice a day, and I often find the disease proceed uninterruptedly to its cure.

"Again, when by the same ordinary course of treatment fever, pain, and swelling have been made to cease entirely, and have suddenly and unexpectedly returned, then I invoke the aid of colchicum, and give it in the same way; and a few doses are commonly enough to dissipate the returning disease, and restore the conditions of health. This is a pure case of relapse. The relapse, however, very seldom reaches the severity of the original attack.

"Now on all such emergencies I have been accustomed to administer the remedy quite alone, uncombined with either alkali or opiate, so that the benefit which has resulted has been without question exclusively due to it; and not only exclusively due to it, but due to it purely as colchicum in virtue of that mode of action, (whatever it may be) which specifically belongs to it. For the cure has followed suddenly, and not waited for any immediate operation of the remedy upon the stomach and bowels."

These same remedies—bleeding, opium, and calomel—must be used when inflammation of the heart is *actually* present; "but bleeding in different modes and measures, mercury directed to a totally different purpose, and opium given with more than one single intention." Some of the circumstances and conditions of actual practice in which we meet with endocarditis or pericarditis, are thus noticed—

"Take a case which you have watched and treated day after day; a case in which day after day you have examined and listened to the chest, fearing and guarding against surprise, and have felt all possible confidence that hitherto the heart is free. In such a case pain arises, distinct and sudden pain in or near the præcordial region; or the heart suddenly begins to beat with excessive impulse; or the heart, as if struck with weakness, suddenly begins to flutter and act irregularly. But withal your ear detects neither bellows-murmur, nor sound of attrition. What then are you to think of the disease, and what to do? You are to conclude that inflammation is begun in the heart, and you are to take measures to subdue it without a moment's delay.

"Pain in the heart; excessive impulse of the heart; irregular action of the heart; any one of these, or any two, or all of them together, coming on in the course of acute rheumatism make inflammation of the heart so nearly certain, that it would be folly to suspend the remedy by waiting for more certainty, and so running the hazard of having a more advanced and less tractable disease to deal with.

"Take another case of rheumatism, in which you have been daily upon the watch for the least sign of evil befalling the heart, and hitherto have found none: no pain; no excess of impulse; no irregular action. In such a case, when you least expect it, you may hear something unusual in the heart. It may be its systolic sound prolonged in an undue degree, or a little harsh, and nothing more. Yet this, if it be really so, even this alone is enough to call for instant measures to subdue inflammation of the heart. For it is almost certain that, by to-morrow, this sound, a little more prolonged, and a little harsher than natural, will become a genuine bellows-murmur, and declare the sure existence of endocarditis.

"Take another case of rheumatism, in which neither præcordial pain or excess of impulse or irregular action have hitherto drawn attention to the heart, nor do they now. But now your ear may begin to admonish you of something belonging to the heart, which was not there the last time you listened: a sound which is no modification of what is found in health, but is entirely new, and unnatural. This sound may be as yet audible in so small a space of the præcordial region, that, of several who have been carefully listening to the patient's chest during the last half hour, one only has happened to light upon it. Yet is it so sure and certain a

5

reality, that no sooner is attention pointed to the spot, than all can hear it, and all have no doubt of it. The sound is an indefinite sound still; each person who hears it likens it to something different. It is not such as can fix disease either upon the endocardium or the pericardium. But coming on in the course of acute rheumatism, it must be taken for an announcement that all is not right in the heart, that it has begun to suffer after the manner to which acute rheumatism renders it obnoxious, and that it is already in a state of inflammation. Therefore the remedy must no longer tarry. Ere a day is gone, this indefinite sound will be changed into an endocardial or exocardial murmur.

"Take yet another case in which, from the absence of all symptoms referable to the heart, you believe it to be hitherto unhurt. You listened perhaps a few hours ago, and still found nothing to excite suspicion. And now listening again you hear the endocardial, or you hear the exocardial, murmur completely formed. These sounds, being the concomitants of acute rheumatism, announce at once inflammation of the heart, and fix its seat either in the membrane that lines it within, or the membrane which invests it without. Here nothing is wanting to the instant and complete disclosure of the disease; and nothing should be wanting to the immediate and efficient treatment of it.

"These are the ways in which I have seen endocarditis or pericarditis begin, when I have witnessed their beginning. Things different in kind, a pain, an impulse, or a sound; things great or small, palpable, or hardly discernible, and never discerned but by the practised sense, have given the first authentic notice of these awful diseases. Therefore the eye, the ear, and every sense and faculty which can convey intelligence of what goes on within a man should be kept upon the watch for these things."

When any of these signs appear, our treatment should be directed to the heart. This *curative* treatment is thus sketched:

"As soon as inflammation is known or suspected to have reached the heart, mercury must be given without delay. Or should mercury be already in use, as a remedy for acute rheumatism, with the intent of obtaining large evacuations from the bowels, it must at once have a new direction given to it. The irritation it has produced within the abdomen must by all means be pacified, and its constitutional impression must now alone be thought of,—that impression, of which salivation is the best evidence, of which (as far as we know) it alone, of all remedial substances, is properly and exclusively capable, and which under favorable circumstances, is largely counteractive of inflammation.

"But, inasmuch as that impression of mercury, or salivation, which is the best evidence of it, cannot be commanded within any given time, you must be content to administer it in the manner calculated for this purpose, and wait the event. For no judgment can be formed of its curative effect upon the disease until salivation arrives. And it may arrive in a day or two, or not until after several days, or after a week, or after several weeks, or it may not arrive at all.

"You must give mercury, then, and wait until salivation come to tell you what it has done for the disease, or until it is fair to conclude there will be no salivation. And it does, I confess, require strong confidence in this great remedy, thus to give it, and thus to wait for its ultimate effect, when in the meantime it displays no proximate or intermediate effect as an earnest of its curative operation.

"With respect to venesection, if in acute rheumatism, any of those symptoms referable to the heart are present, which have been already mentioned, auscultatory or non-auscultatory, and especially if they have arisen under your own observation, or, though not under your own observation, if they be now present, and you have reason to believe that they have *recently* arisen, then, should the pulse be found to have even a notable degree of that hardness which is deemed inflammatory, blood must be taken from the arm. Should there be any doubt about venesection, any misgiving whether the inflammatory hardness of the pulse is quite enough to require it, let it be employed nevertheless. There is greater hazard in omitting it wrongfully, than in practising it wrongfully.

"But with the same amount of vascular action evidenced by the state of the pulse, in a case of mere rheumatism, in which the heart was not affected, venesection would be neither necessary nor proper.

"Again, if in acute rheumatism, with symptoms referable to the heart and those recently declared, fever and vascular action run very high, and there be extreme fulness and hardness of the pulse, a copious venesection should be practised. And the same should be repeated, were the hardness and fulness of the pulse found not to yield. And repeated again and again, until the hardness and fulness of the pulse were much abated.

"With all this fever and inflammatory action, and all this hardness and fulness of the pulse, had the case been one of mere rheumatism, and the heart unaffected, venesection would have been properly employed, indeed once or even twice, but it would not have been carried further for reasons formerly assigned. Now, however, more danger is to be feared from the progress of inflammation in the heart, than from any shock imparted to the nervous system by loss of blood.

"But though the disease be inflammation, and the organ inflamed be a vital organ, even the heart, bleeding must have a limit; for bleeding cannot alone be trusted to cure the disease. Mercury must become its auxiliary. And if for no other reason than to obtain from the mercury a fuller curative operation, bleeding must have a limit.

"Looking, as I do, mainly to mercury to save life or to save the organ, I am constantly careful in the management of every case to do everything to aid, and nothing to hinder its curative operation. Especially in the use of venesection, I bear this in mind; for sure I am that loss of blood can both aid and hinder it according to its amount.

"In men of florid aspect and full blood-vessels, though *bleeding* has not been needed for its own sake, yet has it oftentimes been *moderately* used for expediting the sensible effects of mercury. And the sensible effects thus induced have been at the same time curative. But, if the body be first made exsanguine by the lancet, you may gain the *sensible* effects of mercury, and lose the curative; for the two do not of necessity go together.

"As mercury can be less trusted for its antiphlogistic power in those who either naturally or from some previous disorder are pale and anæmic, so in the robust and sanguineous if you would have mercury exercise that power it has for the control of inflammation, you must beware of *making* these what the others *are*. You must hold your hand from excessive venesection. In inflammation of the heart you must not first change a fine ruddy countenance into the aspect of a chlorotic girl, and then leave mercury to complete the cure without anxiety or distrust of the event.

"Recollect, then, that in rheumatic inflammation of the heart, whether it be endocarditis or pericarditis, blood-letting by venesection is to be little in one case, and much in another, according to the present force of vascular action throughout the body; and little or much respective to its own proper benefit as a remedy; and little or much respective to its secondary uses in procuring the more effective operation of mercury. And recollect, moreover, the cautionary admonition applicable alike to the management of this and of all diseases in which a large share of the cure is confided to mercury, recollect, not to lessen or to lose its curative operation, either by bleeding too little or bleeding too much; for plethora and anæmia are alike obstructive of it.

"But there are other modes and means of blood-letting besides venesection. There is cupping, and there are leeches.

"Now in rheumatic inflammation of the heart, cupping or leeches, or both, may be needed as auxiliary to venesection; or exclusive of venesection, they alone may be needed and trusted for taking as much blood as the nature of the case requires.

"As it often happens to other organs when they are inflamed, so also to the heart, that when general vascular action has run high and venesection has been employed to reduce it, and *has* reduced it effectually, the local symptoms will remain altogether, or almost, unmitigated. Thus pain and palpitation of the heart, and unnatural sounds, often abide in the same degree, or nearly so, after venesection as before it; and then cupping or leeches come in as effective auxiliaries, and the local bleeding often makes at once an appreciable impression upon what the general bleeding has not touched.

"Again, as in other parts of the body, so in the heart, it is no uncommon thing for inflammation to begin and proceed onwards to destructive disorganisation *without being felt as an inflamation* by the constitution at large. It imparts no special hardness to the pulse, and no extraordinary force to the circulation, yet is it working its own injurious changes locally: palpitation, or pain, or irregular action, or unnatural sounds, declare as much. Here all that blood-letting can compass for the relief of the disease and its symptoms will be attained by cupping, or by leeches alone.

"Now there is a choice between cupping and leeches. One may be a more appropriate remedy than the other in a particular case; and yet I dare hardly trust myself, apart from the patient, to point out what it is that should determine you to prefer one to the other.

"So dexterous are those who are well practised in the art of cupping that they can make their glasses draw a given quantity of blood in almost as short a time as the lancet; whereas leeches are long at work in taking away the quantity you desire. Therefore, upon the principle of requiring in the remedy a force and rate of operation proportionate to the force and rate of the disease, I should say that when the pain or anguish, or by whatever name you may call the distress, immediately referable to the heart, begins suddenly, is at once felt severely, and augments rapidly, then cupping is the remedy; but that, when it comes on by little and little, and increases slowly, and has not reached a great amount, then leeches are the remedy. But leeches are often needed as auxiliary to cupping, just as cupping is to venesection.

"There is another thing worth mentioning as to the use of these remedies. When I have employed cupping in inflammation of the heart, I have been accustomed to have the glasses applied between the left scapula and the vertebral column. Applied upon the præcordial region, they have seemed to me to cause peculiar distress, and to be not without the hazard of doing mischief and violence to the heart in its present condition; for recollect that *now* a slight percussion upon the cartilages of the ribs, or any degree of pressure which shall carry them ever so little downwards towards the heart, will often occasion pain. Besides, blood taken from between the scapula and the spine produces all the relief to the heart which could be expected.

"But we must come nearer yet to the bedside of the patient, and look still more closely into the immediate effect of blood-letting in inflammation of the heart. Its effect, day by day, is the earnest of its ultimate effect. The sensible impression of each venesection, or of each cupping, or of each application of leeches, is a measure of the good they are doing, and of the trust to be reposed in them as remedies. But what is this effect which is to be looked for day by day?

"In inflammation of the lungs or pleura, blood-letting shows its favorable impression by setting free the respiration and diminishing pain. In inflammation of the brain, by abating delirium, or coma, or spasm, by restoring clearness to the sense, and intelligence to the mind; in inflammation of the peritoneum, by softening the tense abdomen, and making it more patient of pressure. And if the heart be inflamed either within or without, any of the several modes of blood-letting will denote itself to be exercising a curative power upon the disease, when it moderates pain, or when it moderates excessive impulse, or when it renders its beats regular again, which were irregular before. Thus it is in the sensations and functions of the heart as of other organs, that we see the benefit which blood-letting is doing, or is ultimately to do, towards the cure of its inflammation.

"Next as to the use of opium. It indeed is needed now that the heart is inflamed, to calm the nervous system, and to abate pain, as it was needed before; and moreover, it is needed for another purpose, viz: to give effect to the operation of mercury in one mode at least of administering it.

"If in aiming to produce salivation you give calomel internally, you must restrain it from running off by the bowels: and this can only be done by opium. Opium must be combined with every dose of calomel, and the quantity of each must be proportioned to the other. Thus, perhaps, we shall generally find ourselves compelled to give more opium for the purpose of keeping the bowels patient under the stimulus of calomel, than we should give merely to soothe the nervous system under the general irritation of the disease. But, as the remedial operation of mercury is not now under consideration, it is unnecessary to say more at present of opium as its adjunct and auxiliary.

"Besides, however, these, its common uses of calming the nervous system, and abating pain and aiding the effect of mercury, opium has its extraordinary uses for extraordinary emergencies. In endocarditis and pericarditis, not always, but most commonly, pain is present; pain of the heart varying in amount, and upon the whole abiding, yet allowing remissions and abatements. But whether the customary pain be present or no, a sudden agony will sometimes seize the heart, and at once the patient will feel and look as if he had received his death -blow. It is like a spasm of the heart. It is no other than a paroxysm of angina pectoris. This terrible seizure attends pericarditis more frequently than endocarditis, and where it has once occurred, it is apt to occur again. Its only remedy, as far as I know, is a large dose of opium. Opium is the only means of rescue during the agony, and the only safeguard against its return."

The author places great reliance on the use of mercury in these affections, chronic as well as acute; and he discusses the subject of its employment at very considerable length. We must pass by all this with the following extract:

"Mercury, as a remedy for inflammation, requires to be administered with as careful an adaptation of its dose to the exigencies of each particular case, as bleeding does of its mode and quantity. The principle of practice is this, to measure the force and rate of the counteractive impression to be produced, whether by blood-letting or by mercury, by the force and rate of the disease. If you have an inflammation of the greatest force and greatest rapidity to deal with and organic changes are taking place from hour to hour, and blood-letting is to be your remedy, its mode must be by venesection, and its quantity must be of large amount; and it may need to be repeated again and again, and at short intervals. The blood-letting must make itself felt with a force and a rapidity which shall exceed the force and rapidity of the disease, otherwise it is no remedy at all. So, too, in the same conditions of inflammation, if mercury is to be your remedy, its dose must be large, and repeated. Calomel must be given in the dose of half a scruple at once, and again after six or eight hours. The patient's life may depend upon his being salivated within a couple of days. The mercury, like the blood-letting, must make itself felt, by its specific effects more forcibly, and more rapidly, than the disease, otherwise it will fail of its counteractive operation.

"But if the inflammation be of little force, and slow in its progress, and its organic changes such as are not visible from hour to hour, or even from day to day, but rather from week to week, then, if your remedy be blood-letting its mode must not be by venesection, but by cupping or leeches, and its quantity must be small, and, if it be repeated, it must be at more distant intervals. For now it is essential to the cure that the counter-impression of the blood-letting should be kept down to the small measure of power, and the slow rate of progress, belonging to the inflammation; otherwise it is no remedy at all. And in like manner, if mercury be your remedy, it mst now be given more sparingly and less frequently. The object is to produce salivation, not as soon as possible, but slowly and without surprise or violence. A grain or two of calomel once or twice a day, or even some milder preparation of mercury, will bring its specific effects to bear upon the disease after many days, but still remedially."

Touching the curability of hypertrophy of the heart, Dr. Latham's experience is opposed to that of many physicians.

He has never yet met with a single instance in which he was satisfied that a cure was effected. In the cases reported as cured, the disease was only counterfeited.

"I am well aware that there is a mock hypertrophy of the heart bearing so close a resemblance to the true, that I should find no fault with you for being taken in by the counterfeit.

"There may be violent impulse of the heart, felt not only in the præcordial region but in every part of the front of the chest upon which you lay your hand; and there may be pain in the heart, and pain and throbbing in the head; and all these may be never absent and often aggravated from time to time by accidental circumstances; and they may continue from first to last for several months, or for several years, and produce in the meanwhile an incapacity of all useful exertion both mental and bodily: all these may be, and yet there be no hypertrophy.

"Impulse of the heart, taken alone, however great and however extensive it may be, is not a sure physical sign of hypertrophy. Hypertrophy indeed cannot exist without excess of impulse, but excess of impulse can exist without hypertrophy. When the impulse of the heart is excessive, and at the same time its sounds are obtuse, muffled and indistinct, and the præcordial region presents a larger space than natural which is dull to percussion, then the signs of hypertrophy are complete. And hypertrophy so sure and unquestionable was never cured within my experience. But when the impulse of the heart is in excess, and at the same time its sounds are as loud and clear as ever, or louder and clearer still, and the whole præcordial region is quite resonant to percussion save the small space which is naturally dull, then the signs of hypertrophy are incomplete. Yet if this be enough to constitute hypertrophy, I have seen and treated it successfully in a hundred instances. But in the meantime I have not thought that I had to do with any such affection or ever claimed the least credit for curing it.

"Cases of mock hypertrophy of the heart are indeed very numerous. Young persons at the prime of life are especially the subjects of it. They are often plethoric and often sedentary, and can assign the origin of their complaint to no particular time and to no particular exciting cause. In them the excessive action of the heart is doubtless owing to a rich and redundant blood; and the cure of their simulated hypertrophy is effected by depletion and abstinence and the gradual exchange of indolent for active habits. These are easy cases to deal with.

"Again, young persons are the subjects of it, but they are often pale and thin and dyspeptic and very sensitive, and inactive from mere debility and nervousness. In them the excessive action of the heart cannot be ascribed precisely to any one thing. The stomach and the nerves and the blood itself are all disordered, and they all are sources from which injurious influences may spring up and travel to the heart; and they all have probably their share in producing the simulated hypertrophy. Being so produced its cure can only be effected by varied methods of treatment and after a long time, and often not until the constitution has undergone some of those changes which belong to stated periods of life.—These are by no means easy cases to deal with.

"Again, young persons are the subjects of it, but they are often neither florid nor pale, neither too full nor too empty of blood. They have no complaint that they can tell you of, and none that you can make out, except an inordinate impulse of the heart; an impulse great enough for any amount of hypertrophy, and constantly present, and admitting of severe aggravation, and ever attended with pain, while the sounds of the heart are still loud and clear, and the præcordial region is still duly resonant."

We close our meager notice here although it is neither adequate to the merits of the author, nor a just measure of our appreciation of them. We commend the book most heartily to our readers, as one of high value, and not less pleasing than valuable.

